# Supplementary material for: Aggregative cycles evolve as a solution to conflicts in social investment
Source: PLoS Comput Biol. 2021 Jan 20;17(1):e1008617. doi: 10.1371/journal.pcbi.1008617 (PMC7850506; doi:10.1371/journal.pcbi.1008617)
Supplement: S1 Text — (PDF) [file pcbi.1008617.s001.pdf]

Aggregative cycles evolve as a solution to conflicts in social investment

Leonardo Miele (mmlm@leeds.ac.uk), Silvia De Monte (silvia.de.monte@bio.ens.psl.eu)

S1 Text

Equilibria of the eco-evolutionary dynamics and linear stability analysis

In this section we describe two equivalent formulations of the resource-consumer dynamics, and we present the linear stability analysis of the model.

The system is fully described by the three variables R, N_F and N_S, denoting respectively: resource, number of fast cells and number of slow cells. Then, the dynamics is given by the following ODES:

$$\frac{dR}{dt} = R\left[r\left(1-\frac{R}{K}\right)-(N_F+N_S)\right] \tag{1}$$

$$\frac{dN_F}{dt} = N_F\left[p_F\,R-d\right] \tag{2}$$

$$\frac{dN_S}{dt} = N_S\left[p_S\,R-d\right], \tag{3}$$

where K is the carrying capacity of the resource and d is the mortality rate of both kinds of cells. The growth rate of each cell type differs because of their reproduction rate, that is proportional to the type's payoff (see main text for the definition of the payoffs).

The system can be equivalently described using total population size and composition. We define $N = N_F + N_S$ the total population, and $x = \frac{N_F}{N}$ the fraction of fast cells. Combining (2) and (3), we

get the dynamics for the total population:

$$\begin{aligned}\frac{dN}{dt} = \frac{dN_F}{dt} + \frac{dN_S}{dt} &= N_F\, p_F\, R + N_S\, p_S\, R - d\,(N_F + N_S) \\ &= N\left[\frac{N_F}{N}\, p_F\, R + \frac{N_S}{N}\, p_S\, R - d\right] \\ &= N\,[x\, p_F\, R + (1-x)\; p_S\, R - d] \\ &= N\,[\bar{p}(x,R)\, R - d]\,, \end{aligned} \tag{4}$$

where in the last line we have used Relation (2) of the main text. Equation (4) states that the total consumer population grows at a rate that is proportional to the average payoff $\bar{p}(x, R)$.

Applying the chain rule for the temporal derivative of the variable x and using (2) and (4), we get the dynamics for the social composition:

$$\begin{aligned}\frac{dx}{dt} = \frac{d}{dt}\left(\frac{N_F}{N}\right) &= \frac{1}{N}\frac{dN_F}{dt} - \frac{N_F}{N^2}\frac{dN}{dt} \\ &= \frac{N_F}{N}\,[p_F\, R - d] - \frac{N_F}{N}\frac{N}{N}\,[\bar{p}(x,R)\, R - d] \\ &= x\,(1-x)\,R\,[p_F(x,R) - p_S(x,R)]\,, \end{aligned} \tag{5}$$

which takes the form of a replicator equation for the cell types fractions.

Eq. 1 of the main text, in terms of the resource density, total population size and fraction of fast cells, is finally given by Eqs. (1), (4) and (5):

$$\begin{aligned}\frac{dR}{dt} &= R\left[r\left(1 - \frac{R}{K}\right) - N\right] \\ \frac{dN}{dt} &= N\,[\bar{p}(x,R)\, R - d] \\ \frac{dx}{dt} &= x\,(1-x)\,R\,[p_F(x,R) - p_S(x,R)] \end{aligned}$$

with:

$$\bar{p}_F(x,R) = \alpha\, R\, \lambda_F$$

$$\bar{p}_S(x,R) = (1 - \alpha\, R)\, x\, \lambda_S,$$

where αR is the probability that a cell is found in isolation, that we assume to depend on the resource availability. This formulation highlights the distinction between the ecological consumer-resource dynamics (given by the equations for R and N), and the evolutionary dynamics describing the competition between the two cell types (given by the replicator equation for x).

In the main text, we considered that this probability varies between zero, when no resource is available, to one, when resources attain the carrying capacity. This is modelled by choosing $\alpha = 1/K$. This choice was driven by simplicity and in order to reduce the number of free parameters. Since the amplitude of the oscillations of the resource depend on all parameters, it indirectly bounds the probability in an interval strictly contained in $[0, 1]$. In S2 Text we consider the more general case when $\alpha \neq 1/K$, but in the following of this section we will keep the same scaling as in the main text.

The average payoff thus takes the form:

$$\bar{p}(x, R) = \left[(\lambda_F - \lambda_S) \frac{R}{K} + \lambda_S \right] x - \left(1 - \frac{R}{K} \right) \lambda_S \, x^2.$$

The dynamical system has two sets of degenerate trivial equilibria $(0, 0, x)$, and $(K, 0, x)$, the latter corresponding to the resource being at its carrying capacity in the absence of consumers.

If $\lambda_F \, K/d > 1$, the system has one fixed point where only fast cells are present:

$$\hat{R}_F = \phi \, K \qquad \hat{N}_F = r \, (1 - \phi) \qquad \hat{x}_F = 1, \tag{6}$$

where for convenience we have defined:

$$\phi = \sqrt{\frac{d}{\lambda_F \, K}}$$

the fraction of the carrying capacity at which the resource is at equilibrium. This composite parameter appears in several expressions derived in the following, demonstrating that the effect of death rate d and of carrying capacity K on the system equilibria and on its evolutionary dynamics are compounded, so that an increase in the former can be compensated by a proportional increase of the latter.

If, furthermore, the condition:

$$\phi\left(1+\frac{\lambda_F}{\lambda_S}\right)<1 \tag{7}$$

is satisfied, then a coexistence equilibrium exists:

$$\hat{R}=\phi\,K \qquad \hat{N}=r\,(1-\phi) \qquad \hat{x}=\frac{\lambda_F}{\lambda_S}\,\frac{\phi}{1-\phi} \tag{8}$$

where the proportion of fast cells decreases when the level of exploitation by slow cells increases. In order for a coexistence equilibrium to exist, thus, not only the payoff of fast cells needs to be large enough for their population to survive based on the resource available, but slow cells also need to have a sufficiently high 'incentive to cheat', that is their payoff needs to be sufficiently high so that communal living provides sizable advantages. Relation (7) means that slow cells can survive when resources are depleted as a result of consumption by fast cells.

The trivial manifold $(0, 0, x)$ is always a saddle point, corresponding to the fact that in the absence of consumers, the resource will increase. As long as $\lambda_F \leq d/K$, the equilibria with $R = K$, $N = 0$ are stable for any x, that is the consumers will go extinct and the resource reach its carrying capacity.

The fast-only equilibrium Eq. 6 is stable as long as no positive coexistence equilibrium Eq. 8 exists. The leading eigenvalue of the Jacobian matrix in the fast-only equilibrium:

$$J(\hat{R}_F,\hat{N}_F,\hat{x}_F)=\begin{pmatrix} -r\;\phi & -\phi\,K & 0 \\ 2\,\lambda_F\,r\,\phi\;(1-\phi) & 0 & r\,\phi\,K\,(1-\phi)[\lambda_F\phi+\lambda_S(\phi-1)] \\ 0 & 0 & -\phi\,K[\lambda_F\phi+\lambda_S(\phi-1)] \end{pmatrix} \tag{9}$$

becomes positive when relation (7) is satisfied, that is when the coexistence equilibrium exists. In this case, this equilibrium is a saddle point.

The eigenvalues of the Jacobian matrix $\hat{J}$ in the internal equilibrium $(\hat{R}, \hat{N}, \hat{x})$ is:

$$\hat{J} = \begin{pmatrix} -r\,\phi & -\phi\,K & 0 \\ \frac{r\lambda_F\,\phi}{\lambda_S\,(1-\phi)}\left[\lambda_S + \phi\,(\lambda_F - 3\lambda_S + 2\lambda_S\phi)\right] & 0 & r\phi\,(1-\phi)\,K\left[\lambda_S - (\lambda_F + \lambda_S)\,\phi\right] \\ \frac{\lambda_F^2\,\phi^2}{\lambda_S^2\,(\phi-1)^3}\left[(\lambda_F + \lambda_S)\,\phi - \lambda_S\right] & 0 & \frac{\lambda_F\,\phi^2\,K}{\lambda_S\,(\phi-1)}\left[\lambda_S - (\lambda_F + \lambda_S)\,\phi\right] \end{pmatrix}$$

can be numerically evaluated as a function of the parameters, and the equilibrium can be shown to be stable close to the transcritical bifurcation that generates the coexistence equilibrium, and successively to bifurcate into an unstable focus.

This Hopf bifurcation of the coexistence equilibrium occurs when the characteristic polynomial of the Jacobian matrix transitions from having three real solutions to having one real and two complex-conjugate solutions. The bifurcation condition is thus that two roots of the third-degree characteristic polynomial are simultaneously null. We thank Alice l'Huillier and Bertrand Maury for pointing out that we could use Cardano's method to numerically compute the boundary of the region in a 2-dimensional parameter space were the system oscillates.

If we fix the parameters $d = K = r = 1$, then the Jacobian matrix as a function of the evolutionary parameters, evaluated at the coexistence equilibrium, reads:

$$\hat{J} = \begin{pmatrix} -\frac{1}{\sqrt{\lambda_F}} & -\frac{1}{\sqrt{\lambda_F}} & 0 \\ \frac{1}{\lambda_S(\sqrt{\lambda_F}-1)}(2\lambda_s + \lambda_S\lambda_F - 3\lambda_S\sqrt{\lambda_F} + \lambda_F^{3/2}) & 0 & \frac{1-\sqrt{\lambda_F}}{\lambda_F^{3/2}}(\lambda_S + \lambda_F - \lambda_S\sqrt{\lambda_F}) \\ \frac{\lambda_F^2}{\lambda_S^2}\,\frac{(\lambda_S\sqrt{\lambda_F} - \lambda_F - \lambda_S)}{(\sqrt{\lambda_F}-1)^3} & 0 & \frac{\lambda_F}{\lambda_S(\sqrt{\lambda_F}-1)} - 1 \end{pmatrix}$$

The sign of the real part of the eigenvalues λ can now be found as a function of the evolutionary parameters λ_S and λ_F. The secular equation for the Jacobian can be cast in the following form:

$$\mathcal{P}(\lambda) \;=\; \lambda^3 + p\lambda + q,$$

where:

$$p = \frac{(\sqrt{\lambda_F}-1)(\lambda_F-\lambda_S+\lambda_S\sqrt{\lambda_F})}{\sqrt{\lambda_F}\lambda_S(\sqrt{\lambda_F}-1)} - \frac{(\lambda_S-\lambda_F\lambda_S+\lambda_F^{3/2})^2}{3\lambda_F\lambda_S^2(\sqrt{\lambda_F}-1)^2}$$

$$q = \frac{(\lambda_F-\lambda_S+\sqrt{\lambda_F}\lambda_S)(\lambda_S-\lambda_S\lambda_F+\lambda_F^{3/2})}{3\lambda_F\lambda_S^2(\sqrt{\lambda_F}-1)} - \frac{2(\lambda_S-\lambda_F\lambda_S+\lambda_F^{3/2})}{27\lambda_F^{3/2}\lambda_S^3(\sqrt{\lambda_F}-1)^3} - 2\frac{\lambda_F+\lambda_S-\sqrt{\lambda_F}\lambda_S}{\sqrt{\lambda_F}\lambda_S}.$$

All these quantities can be numerically evaluated, given the parameters λ_F and λ_S. Following Cardano's method for the resolution of third order equations, we define the quantity $\Delta(\lambda_F, \lambda_S) = \frac{q^2}{4} + \frac{p^3}{27}$, whose sign determines the nature of the roots. Since $\Delta(\lambda_F, \lambda_S)$ is always positive $\forall \lambda_F, \lambda_S \in [2, 40]$, the Jacobian has one real and two complex conjugate eigenvalues, which we call $\Lambda_{\mathcal{R}}$ and $\Lambda_{\mathcal{C}_{1,2}}$, respectively. $\Lambda_{\mathcal{R}}$ is always negative in the region of the parameters investigated. The real part of $\Lambda_{\mathcal{C}}$, instead, can be either positive or negative. The numerical bifurcation curve, that is the solution of $Re(\Lambda_{\mathcal{C}}) = 0$, is displayed as a white line in S5 Fig. Along this line, the system undergoes a supercritical Hopf bifurcation (Ott 2002), whereby a stable focus gives rise to an unstable focus and a stable limit cycle of frequency $Im(\Lambda_{\mathcal{C}})$.

References

Ott, Edward (2002). *Chaos in dynamical systems.* Cambridge university press.
