# Supplementary material for: Aggregative cycles evolve as a solution to conflicts in social investment
Source: PLoS Comput Biol. 2021 Jan 20;17(1):e1008617. doi: 10.1371/journal.pcbi.1008617 (PMC7850506; doi:10.1371/journal.pcbi.1008617)
Supplement: S2 Text — (PDF) [file pcbi.1008617.s002.pdf]

Aggregative cycles evolve as a solution to conflicts in social investment

Leonardo Miele (mmlm@leeds.ac.uk), Silvia De Monte (silvia.de.monte@bio.ens.psl.eu)

S2 Text

Bounded probability of remaining alone

When the probability α of cells remaining alone is different from $1/K$, cells cannot avoid being in groups even when resources are very abundant. Numerical estimations of the bifurcation boundary (Fig 1) indicate that the qualitative structure of the bifurcation diagram remains unchanged, but the bifurcation boundary displaces towards higher λ_F as the probability of aggregation decreases. This means that, somewhat counter-intuitively, oscillatory solutions emerge more easily when cells have less opportunity to group. This is a consequence of the fact that increased grouping forces an additional load that free-riders impose to the collective function. The total number of cells is thus maintained low, and resources high. The coupling between resource-consumer ecology and population composition can however be re-established if the population as a whole can achieve faster growth by being more efficient in solitary feeding, which occurs for high λ_F. Interestingly, the role of the social exploitation parameter λ_S is almost unchanged in the region when oscillations are possible.

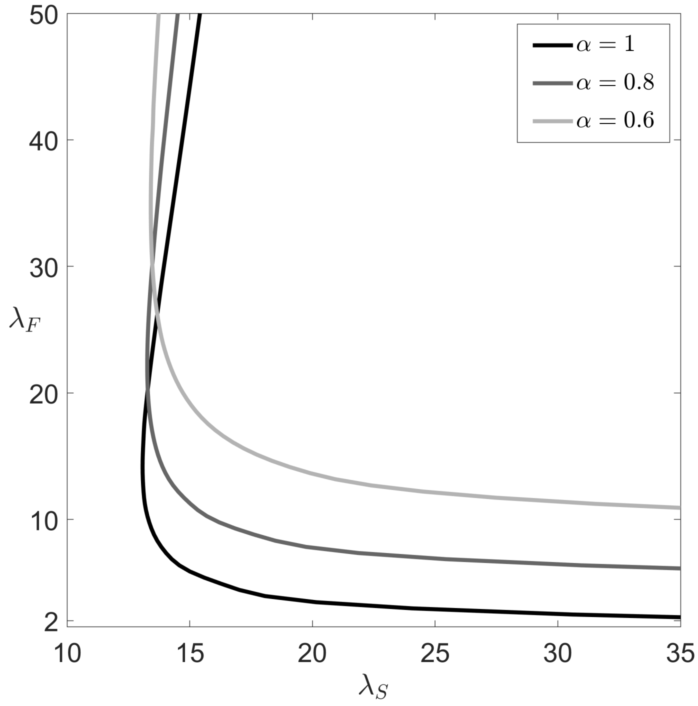

Fig 1: **Dependence of the bifurcation diagram on the probability to aggregate.** As the maximum probability of being in the solitary state decreases, the region when oscillatory behaviour occurs displaces towards higher λ_F.
