# Supplementary material for: Aggregative cycles evolve as a solution to conflicts in social investment
Source: PLoS Comput Biol. 2021 Jan 20;17(1):e1008617. doi: 10.1371/journal.pcbi.1008617 (PMC7850506; doi:10.1371/journal.pcbi.1008617)
Supplement: S3 Text — (PDF) [file pcbi.1008617.s003.pdf]

Aggregative cycles evolve as a solution to conflicts in social investment

Leonardo Miele (mmlm@leeds.ac.uk), Silvia De Monte (silvia.de.monte@bio.ens.psl.eu)

S3 Text

Linear stability analysis of the purely ecological dynamics

Purely ecological equations correspond to the neutral case when both slow and fast types have the same payoff, and the social composition of the population is fixed to the value x_0 for which fast and slow strategies have equal payoff, that is:

$$x_0 = \frac{\lambda_F}{\lambda_S} \frac{1}{\frac{K}{R} - 1}.$$

Consequently, the average payoff $\bar{p}$ will be equal to the fast cells payoff:

$$\bar{p}(R) = p_F(R) = \frac{R}{K} \lambda_F.$$

Then, the purely ecological dynamics is given by the two equations governing the temporal variation of resource and consumer densities:

$$\frac{dR}{dt} = R\left[r\,(1 - \frac{R}{K}) - N\right]$$
$$\frac{dN}{dt} = N\left(\frac{\lambda_F}{K} R^2 - d\right).$$

The corresponding coexistence fixed point $(\hat{R}_{eco}; \hat{N}_{eco})$ is then:

$$\hat{R}_{eco} = \phi\, K$$

$$\hat{N}_{eco} = r(1 - \phi).$$

The Jacobian of the ecological system is thus:

$$J = \begin{pmatrix} r(1-2\frac{R}{K}) - N & -R \\ 2\frac{\lambda_F}{K}\, N\, R & \frac{\lambda_F}{K}\, R^2 - d \end{pmatrix}.$$

By evaluating J at the fixed point we get:

$$\hat{J}_{eco} = \begin{pmatrix} -r\phi & -\phi\, K \\ 2\lambda_F\, r(1-\phi) & 0 \end{pmatrix}$$

According to the Jury conditions, the fixed point will be locally asymptotically stable if $Tr(\hat{J}_{ec}) < 0$ and $Det\,(\hat{J}_{ec}) > 0$ (Strogatz 2018). Here we have:

$$Tr\,\hat{J}_{eco} = -r\phi < 0$$

$$Det\,\hat{J}_{eco} = 2\lambda_F\, K\, r(1-\phi)\phi > 0$$

as long as such a coexistence equilibrium exists (i.e. when $\phi < 1$). Hence, the coexistence fixed point of the ecological module is always asymptotically stable for each value of the single-cell payoffs parameters λ_F, λ_S.

References

Strogatz, Steven H (2018). *Nonlinear dynamics and chaos: with applications to physics, biology, chemistry, and engineering.* CRC Press.
