# Supplementary material for: Aggregative cycles evolve as a solution to conflicts in social investment
Source: PLoS Comput Biol. 2021 Jan 20;17(1):e1008617. doi: 10.1371/journal.pcbi.1008617 (PMC7850506; doi:10.1371/journal.pcbi.1008617)
Supplement: S4 Text — (PDF) [file pcbi.1008617.s004.pdf]

Aggregative cycles evolve as a solution to conflicts in social investment

Leonardo Miele (mmlm@leeds.ac.uk), Silvia De Monte (silvia.de.monte@bio.ens.psl.eu)

S4 Text

Adaptive Dynamics

In the framework of adaptive dynamics, we consider an infinitely large population where slow resident cells are characterized by a level λ_S^* of social exploitation. We assume that a mutation induces a small random phenotypic variation, generating a mutant sub-population of slow cells with exploitation level λ_S close to λ_S^*, i.e. $|\lambda_S - \lambda_S^*| \ll 1$. The eco-evolutionary dynamics in the presence of a population of mutant slow cells is:

$$\frac{dR}{dt} = R\left[r\,(1 - \frac{R}{K}) - N\right] \tag{1}$$

$$\frac{dN}{dt} = N\,[\bar{p}(R,x,y,z)\,R - d] \tag{2}$$

$$\frac{dx}{dt} = x\,R\left[\lambda_F\,\frac{R}{K} - \bar{p}(R,x,y,z)\right] \tag{3}$$

$$\frac{dy}{dt} = y\,R\left[\lambda_S^*\left(1 - \frac{R}{K}\right)x - \bar{p}(R,x,y,z)\right] \tag{4}$$

$$\frac{dz}{dt} = z\,R\big[\lambda_S\left(1 - \frac{R}{K}\right)x - \bar{p}(R,x,y,z)\big] \tag{5}$$

where x, y, z (such that $x + y + z = 1$) are the frequencies of respectively fast and slow resident and slow mutant cells. The average payoff $\bar{p}$ now reads:

$$\bar{p}(R,x,y,z) = \lambda_F\,\frac{R}{K}\,x + (\lambda_S^*\,y + \lambda_S\,z)\left(1 - \frac{R}{K}\right)x.$$

Adaptive dynamics assumes that a phenotypic mutation is initially carried by an infinitesimally small fraction of the population, that is the continuous limit for a mutation occurring in one individual of

a large population. The invasion fitness S is then given by the per capita growth rate of the rare mutant into the resident population. If the population is at equilibrium, this can be computed by linear stability analysis of the equilibrium corresponding to the case when the population is solely composed of residents $(\hat{R}; \hat{N}; \hat{x}; 1-\hat{x}; 0)$:

$$S(\lambda^*, \lambda) := \frac{\dot{z}}{z}\Big|_{(\hat{R};\hat{N};\hat{x};1-\hat{x};0)} = \hat{R}\left[\lambda_S\left(1-\frac{\hat{R}}{K}\right)\hat{x} - \bar{p}(\hat{R}, \hat{x}, 1-\hat{x}, 0)\right].$$

At such equilibrium, from Eq. (4) follows:

$$\bar{p}(\hat{R}, \hat{x}, 1-\hat{x}, 0) = \lambda_S^*\left(1-\frac{\hat{R}}{K}\right)\hat{x}, \tag{6}$$

that substituted in Eq. (5) yields:

$$S(\lambda^*, \lambda) = \hat{R}\left(\lambda_S - \lambda_S^*\right)\left(1-\frac{\hat{R}}{K}\right)\hat{x}.$$

By equating the average payoff of Eq. (6) to that obtained from Eq. (3):

$$\bar{p}(\hat{R}, \hat{x}, 1-\hat{x}, 0) = \lambda_F\frac{\hat{R}}{K},$$

and solving for $\left(1-\frac{\hat{R}}{K}\right)\hat{x}$, we can express the invasion fitness as function of $\hat{R}$:

$$S(\lambda^*, \lambda) = \left(\lambda_S - \lambda_S^*\right)\frac{\lambda_F}{\lambda_S^*}\frac{\hat{R}^2}{K}.$$

Finally, substituting the equilibrium value of $\hat{R}$ (Eq. (8) in S1 Text), we obtain Eq. (4) of the main text:

$$S(\lambda_S, \lambda_S^*) = d\,\frac{\lambda_S - \lambda_S^*}{\lambda_S^*}.$$

The derivative of S with respect to the evolving trait λ_S specifies the fate of the invasion of a mutant whose phenotype has a given distance from the resident (Brännström et al. 2013). Since in our case such derivative:

$$\frac{\partial S}{\partial \lambda_S} = \frac{d}{\lambda_S^*}$$

is positive whenever there is a coexistence equilibrium, a mutant with $\lambda_S > \lambda_S^*$ will eventually invade the population and become the new resident, replacing the previous one. On the other hand, a mutant with $\lambda_S < \lambda_S^*$ would decrease in frequency over time, and go extinct without affecting the resident population composition.

Such analytical results were obtained when the interior equilibrium fixed point exists and it is stable. Numerical integration shows that, when the system's attractor is a limit cycle, the invasion fitness maintains the same scaling as for the equilibrium case and that when it starts invading, the mutant substitutes the resident. We computed numerically the rate of divergence of z after a subpopulation of slow individuals with trait λ_S^* was initialized at an initial frequency $z(0) = 10^{-10}$. The dominant Lyapunov exponent was estimated by linearly fitting $\ln(z(t)/z(0))$ for $0 < t < t_{max}$. Since the trajectory oscillates (the unstable manifold of the limit cycle is not parallel to the z axis), t_{max} has been chosen sufficiently large ($t_{max} = 2000$) so that those oscillations are averaged-out. We checked that the same results were obtained by computing the average rate of divergence, and that, when the system has a stable equilibrium, the numerical results matched the analytic calculation of the invasion fitness.

References

Brännström, Åke, Jacob Johansson, and Niels von Festenberg (2013). "The hitchhiker's guide to adaptive dynamics". In: *Games* 4.3, pp. 304–328.
