# Supplementary figures and images for: Aggregative cycles evolve as a solution to conflicts in social investment
Source: PLoS Comput Biol. 2021 Jan 20;17(1):e1008617. doi: 10.1371/journal.pcbi.1008617 (PMC7850506; doi:10.1371/journal.pcbi.1008617)

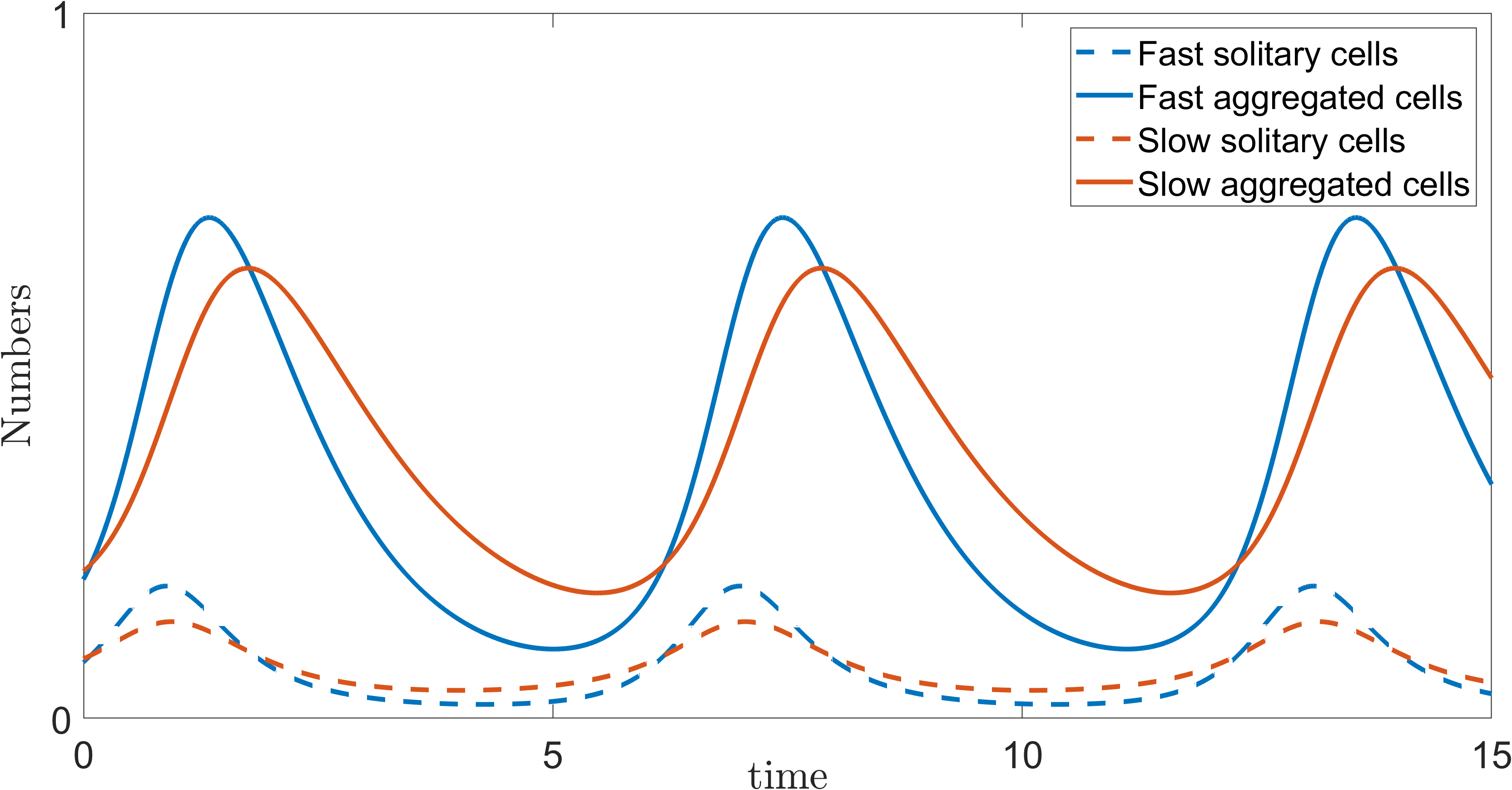

Supplement: S1 Fig — The numbers of fast and slow cells found in the solitary and in the aggregated state oscillate in time along a life-like cycle. These quantities are computed along a limit cycle solution of the eco-evolutionary dynamical system, for the same parameter values as Fig 2 of the main text. (TIFF) [file pcbi.1008617.s005.tiff]

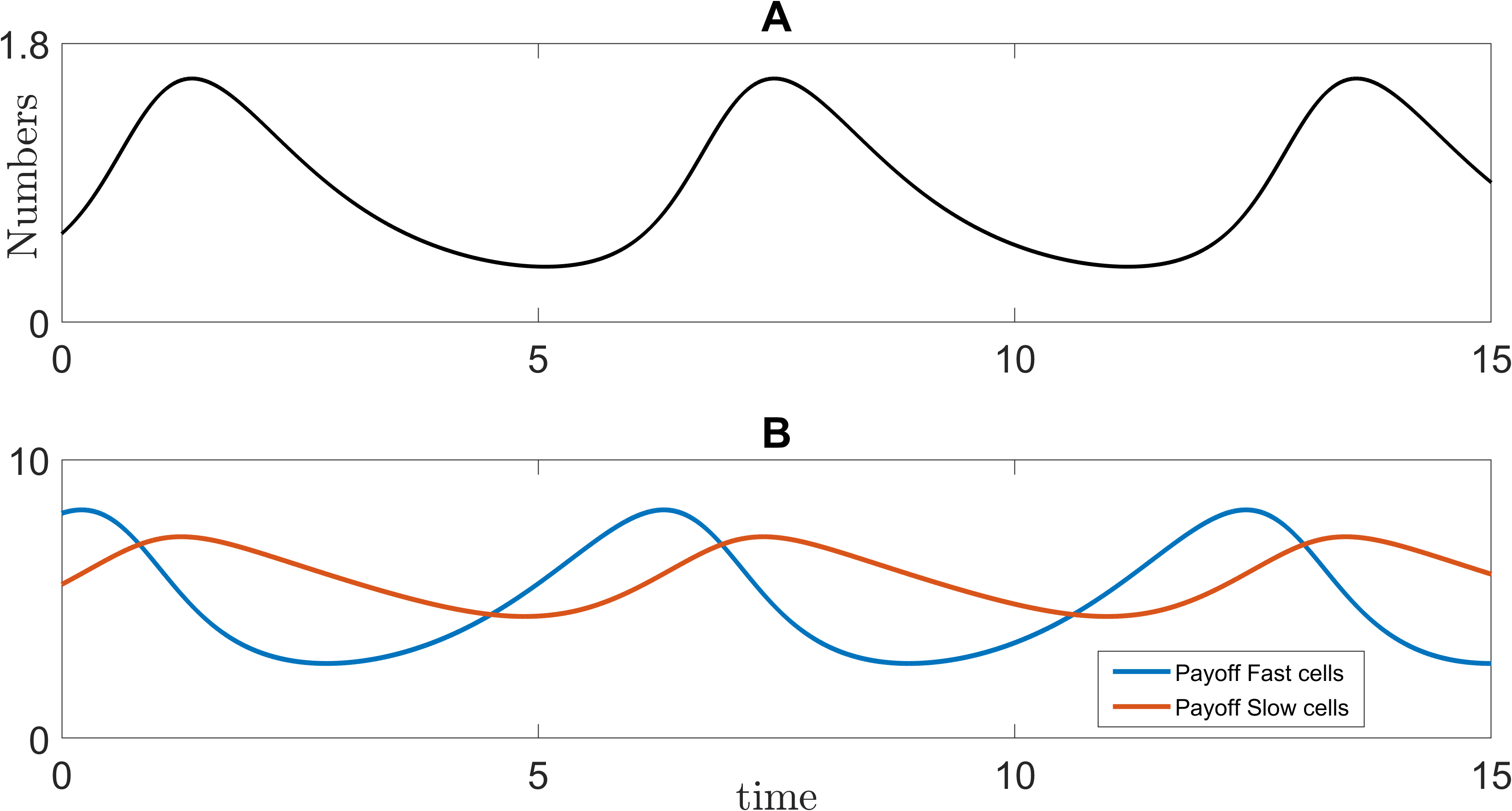

Supplement: S2 Fig — The payoffs of the two strategies oscillate in time as a consequence of the variations in the fraction of aggregated cells and in group composition. These quantities are computed along a limit cycle solution of the eco-evolutionary dynamical system, for the same parameter values as Fig 2 of the main text. (TIFF) [file pcbi.1008617.s006.tiff]

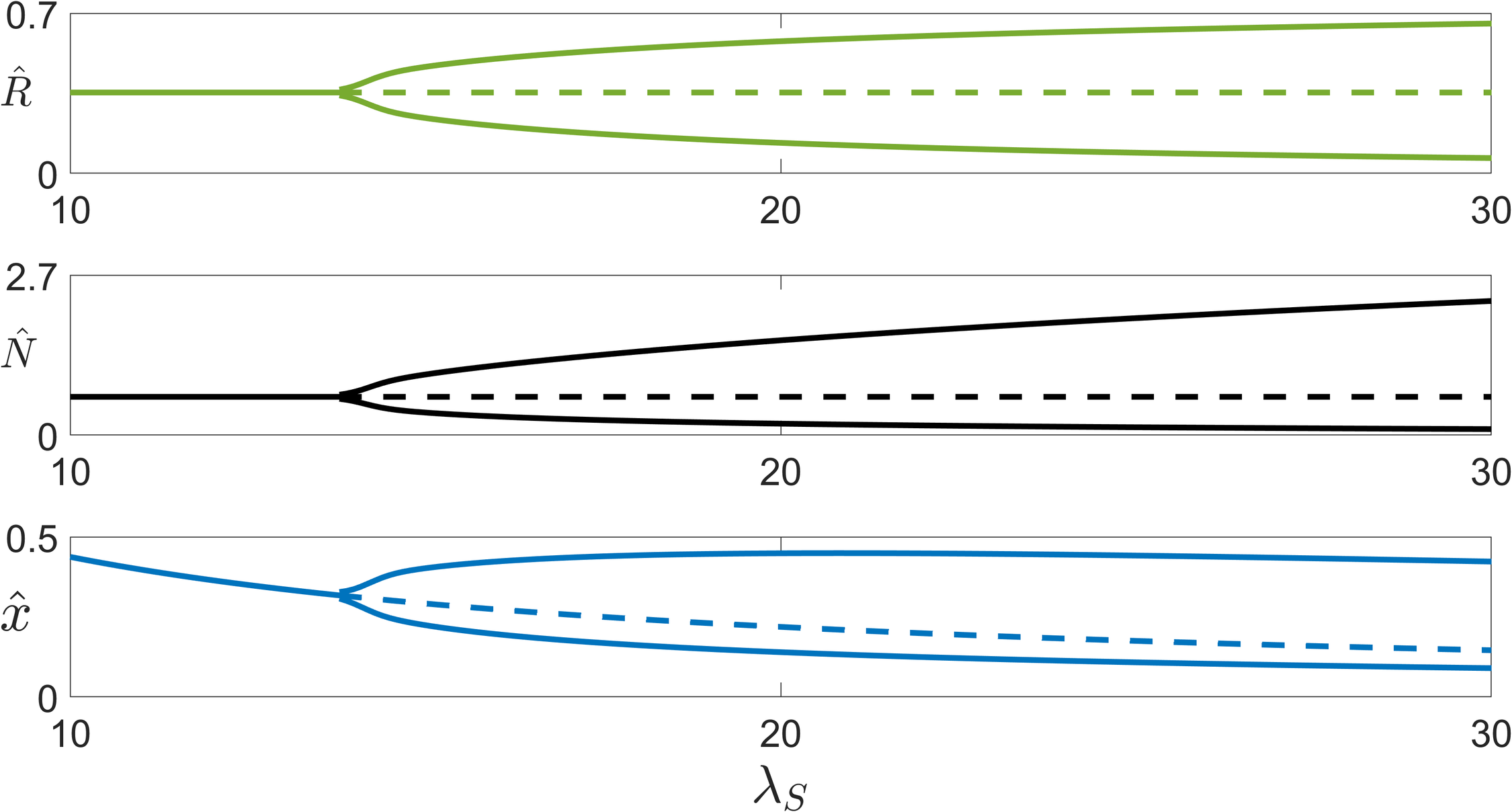

Supplement: S3 Fig — Bifurcation diagrams of the three state variables as a function of the exploitation parameter λS (remaining parameters as in Fig 2 of the main text). Continuous lines indicate the stable coexistence equilibrium and the stable limit cycle, the dashed line indicates the unstable equilibrium. The transition from the coexistence equilibrium point to the limit cycle occurs through a supercritical Hopf bifurcation. The parameter values that identify this bifurcation are numerically computed as explained in S1 Text and are illustrated by the white line in Fig 3 of the main text and S5 Fig. (TIF) [file pcbi.1008617.s007.tif]

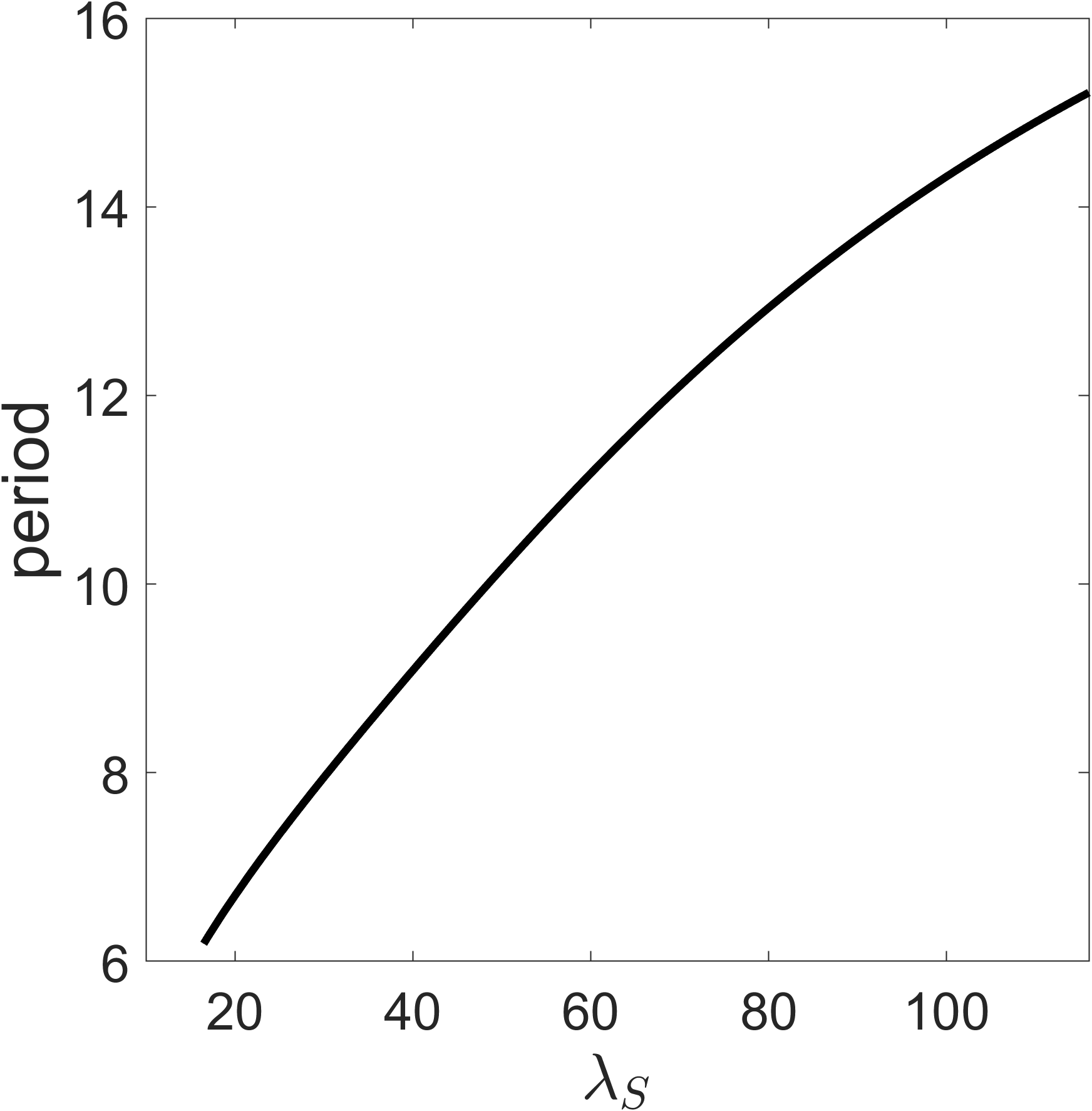

Supplement: S4 Fig — The period of the eco-evolutionary limit cycle increases a function of the exploitation level λS (remaining parameters as in Fig 2 of the main text). The fact that the period does not diverge indicates that an increase in exploitation level does not drive the system towards a global bifurcation that would go undetected by the local analysis of the equilibria we performed. (TIF) [file pcbi.1008617.s008.tif]

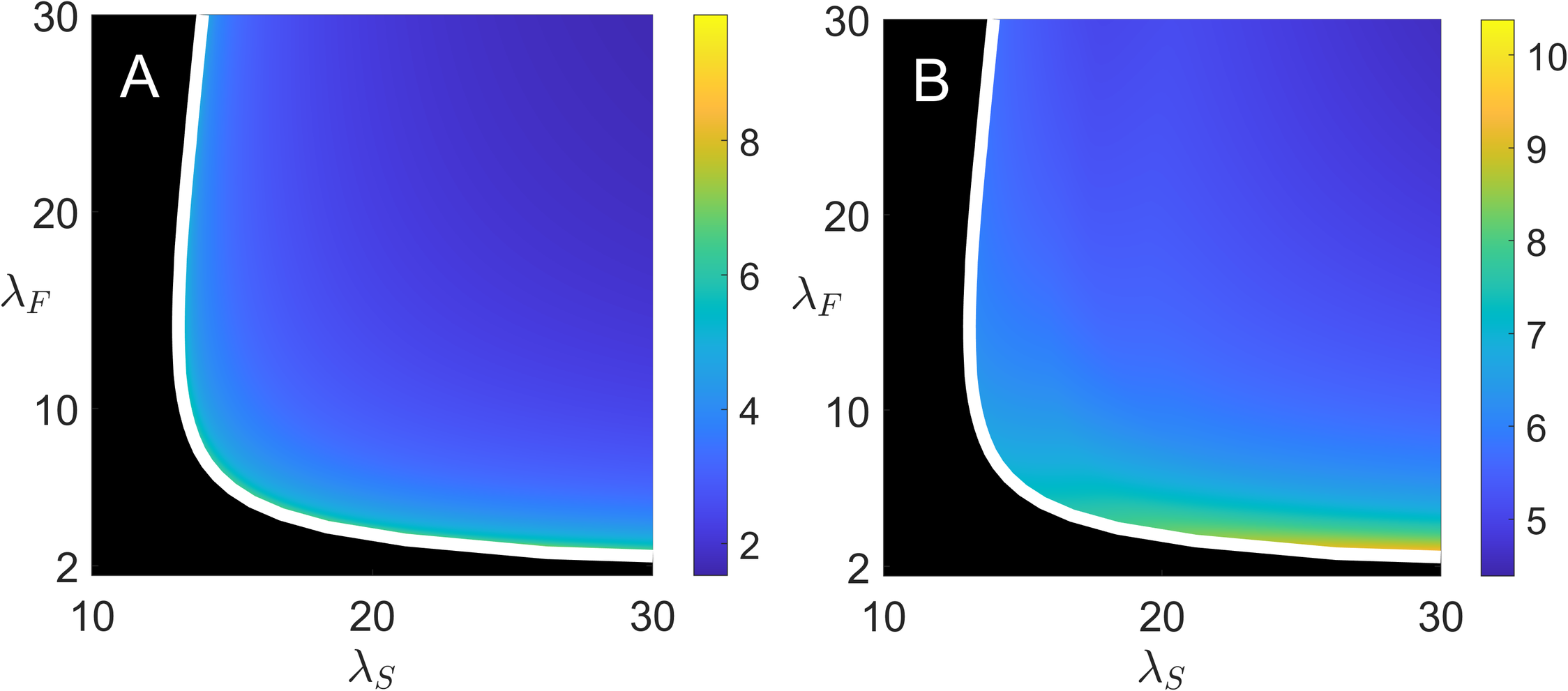

Supplement: S5 Fig — Bifurcation diagrams with respect to the parameters λS and λF (remaining parameters as in Fig 2 of the main text) superimposed to: A) the numerically obtained heatmap of the ratio between the period of the limit cycle and the fastest timescale of the demographic dynamics, computed as the maximum value of the population growth rate along the limit cycle; B) the numerically obtained heatmap of the ratio between the period of the limit cycle and the mean timescale of the demographic dynamics, computed as the average value of the population growth rate along the limit cycle. The white lines are the bifurcation curve analytically derived in S1 Text. The ratio being consistently larger than one indicates that the eco-evolutionary dynamics (thus the cycle of aggregation) occurs on a time scale that is slower than that of cell-level reproduction. (TIF) [file pcbi.1008617.s009.tif]
