# Supplementary material for: Aggregative cycles evolve as a solution to conflicts in social investment
Source: PLoS Comput Biol. 2021 Jan 20;17(1):e1008617. doi: 10.1371/journal.pcbi.1008617 (PMC7850506; doi:10.1371/journal.pcbi.1008617)
Supplement: Attachment — Submitted filename: Response to reviewers.pdf [file pcbi.1008617.s011.pdf]

Dear Dr. Tarnita,

we thank you and the two reviewers for the constructive comments on the manuscript.

We are delighted to hear that you appreciated our approach.

We realized that several points were unclear, both in the assumptions of the model and in the interpretation of the results.

We have therefore extensively reformulated the introduction and the discussion in order to explain:

1. our choice of motility as the trait differentiating the two populations

2. the specific features of aggregative life cycles that we hope our model can account for, and those that would require an extension of the model.

3. the relation with literature on Dictyostelium, that was of course the organism on the back of our minds from the beginning.

We have also introduced more punctual changes in the results section to address the relevant and insightful comments of the reviewers.

The main changes are highlighted in colour, and referenced them in the responses below.

We feel that the manuscript has substantially improved, and we hope you will also find it more to the point.

With best regards,

Leonardo Miele and Silvia De Monte

Reviewer 2

This paper concerns a topic that has recently seen increased interest: the evolution of multicellular life cycles. The authors build a model of a cell population that consumes a resource and forms aggregations when resources are low. Cells exist with different motilities: there is a "fast" type that is successful when solitary, and a "slow" type that is successful when it is in group, through the exploitation of "fast" group members. The authors show that fast and slow types can coexist, and, moreover, that the model can give rise to oscillatory behavior in which the cycles between abundant resources and mostly fast, solitary cells on the one hand, and scarce resources and mostly slow, grouped cells on the other. These oscillations are interpreted population as a mechanism for the eco-evolutionary emergence of aggregative multicellular life cycles.
This is an elegant and creative study, in which the authors use a simple, analytically tractable model to study how social exploitation affects population dynamics. The theoretical result that in a population of microbes that engage in collective behavior (i.e., collective motion), the evolution of social exploitation can lead to population-wide fluctuations is novel and interesting. The paper takes a deliberate approach in which there is no way for exploited cells to circumvent social exploitation. Exploitation of fast cells by slow cells is inevitable, which allows the authors to study the ecological consequences of such exploitation: as exploitation gets more and more severe, population-wide fluctuations emerge, meaning that resource depletion and group formation, followed by resource recovery and return to the solitary stage, become synchronized at the

population level. Most of the results are obtained analytically.
My main concerns are with the presentation and the interpretation of the results. In terms of presentation, the figures can be improved (specific suggestions below). In terms of interpretation, I am concerned with the interpretation of population-wide fluctuations as life cycles. I don't think the term life cycle applies here, and I don't think the authors' model captures the evolutionary emergence of a life cycle.
I have collected some specific comments below. These develop the concerns mentioned above and contain numerous suggestions that I hope will help the authors in improving the paper.

Reply: We are very grateful to the reviewer for their appreciation of our work and for the very useful comments. In combination with the reactions of Reviewer 3, they prompted us to rethink how to convey the main message of our paper, notably on the nature of the eco-evolutionary cycles.

1. The authors use the term "life cycle" to describe population-wide fluctuations of fast and slow cells, as well as the resource. There is no genealogical relationship between fast and slow cells. In the absence of such a genealogical relationship, it seems inappropriate to apply the term "life cycle" here. I don't know of examples in the literature where the term "life cycle" is used this liberally, and I am worried that this departure from the common meaning of the term would lead to confusion.

Reply: We had decided to focus on the dynamical aspect of aggregative life cycles, notably the existence of alternating phases of aggregation and dispersal, that result from evolution of social conflicts in heterogenous groups. Despite models almost invariably assuming a regular occurrence of aggregation (at the end of which fitness of the multicellular phase is evaluated), followed by a dispersal event, the recurrent nature of aggregative life cycles is in our view far from trivial. Moreover, we focused on the emergence of a collective-level timescale, rather than on the phenotypic changes associated to a life cycle, because several models and experiments have shown that phenotypic switches can evolve once an alternating selective pressure is imposed on the population.
However, we agree that the transition between genetically to phenotypically diverse aggregates is not obvious, as also commented by Reviewer 3.
Accordingly, we changed the title in 'Aggregative cycles evolve as a solution to conflicts in motility investment'. In the results we refer to the cycles as 'life-like' cycles. We specify that these are cycles involving population composition and structure, whose temporal arrangement is reminiscent of aggregative life cycles.
In the discussion (lines 292-298), we address the possible role of these cycles in scaffolding the evolution of plastic motility phenotypes.

2. The model builds in the assumption that, depending on resource abundance, cells are solitary or belong to groups. I think of this setup as a dynamic equilibrium between cells and groups, in which groups form by aggregation (when resource levels drop), but groups also can give rise

to single cells (when resource levels increase). These continuing dynamics are also necessary to ensure that group composition reflects the population composition of fast and slow cells, even while in groups it is only the slow cells that reproduce. The existence of these group dynamics implies that there already exists a life cycle at the very beginning of the model, which depends on the rates at which cells join and leave aggregates. In line 25, the authors write that they address "the evolutionary origin of ... life cycles from cellular populations that initially had an essentially unicellular lifestyle"—saying "essentially unicellular" seems inappropriate. I would say that the authors' model captures that social exploitation can lead to population-wide fluctuations in the context of a pre-existing primitive multicellular life cycle, not the de novo emergence of such a multicellular life cycle. In fact, I don't think a model that accounts for population dynamics but not for the dynamics of group formation and group propagation can say too much about the emergence of a multicellular life cycle. All this is not meant to invalidate the authors' results; I think the model as constructed is elegant, cleverly designed in the way it captures important aspects of the biology while still being simple enough to be analytically tractable, and I think the main result is interesting. But I don't think it should be framed as the emergence of a life cycle.

Reply: We disagree that a cycle is present from the beginning. In the stable coexistence regime, no time scale is associated to the aggregation-dissociation dynamics, nor resources vary in time. One can think of it as cells bumping into each other all the time, and reproducing either in isolation or in ephemeral clusters, that form and dissolve even when the partition between the two aggregation states is constant. The population-level oscillations introduce a timescale associated with the recurrence of the state when most cells belong to groups. Population size, composition, and resource get thus synchronized with specific phase differences, so that the cycle consists of a succession of phases reminding those of aggregative life cycles. The fact that life-like cycles last more than one cell generation is another feature that makes them relevant when one looks at collective functions of the aggregates, that are typically (but not in our model) evaluated at the moment of collective-level reproduction, thus on a slower time scale than that of cell division. The collective timescale can be set either by an extrinsic decision as to when the collective is 'mature' (e.g. by a circadian rhythm), or can be linked to the ecology of the population (possibly viewed also as development of the multicellular structure). It is such intrinsic timescale that we want to explain.
We agree however that the model would gain in realism if we had a microscopic counterpart that connected population-level rates to individual-level interactions. We are currently working on developing such a model, but it turns out that even in active matter physics, the field where more advances have been made in studying the collective behaviour of moving particles, there are very few (and recent) studies of how binary mixtures sort.
Based on this and other comments, we have extensively reformulated the manuscript to clarify the focus of our investigation and our assumptions (see below for more precise points).

3. On a related note, I'm curious about the following. Say I build the same model as the authors, but with just a single cell type (no social exploitation), but I make slightly different assumptions so that a limit cycle emerges (this is relatively straightforward in a producer-consumer

model, for example, by changing the functional response). Would the authors also interpret this as the emergence of a multicellular life cycle, because it leads to population fluctuations in the fraction of cells that belong to groups?

Reply: What we interpret as "life-like cycles" are the coupled oscillations in the aggregation state and in population composition. Single-species models might produce oscillations in the aggregation state coupled to population size (though we are not aware of any that looked at this aspect), however they would lack an essential component of aggregative life cycles, their being prone to conflicts among cells that contribute differently to the multicellular phase.

4. In Figure 2, please have the y axes start at 0, and, for the variable x, have it end at 1. Extend the horizontal axis so that more than a single oscillation is shown. Please also plot the fraction of cells in aggregates (SF1a), as well as the payoffs of fast and slow cells. I am confused by SF1B,C. For example, according to the caption, SF1C shows the conditional probability that a solitary cell is fast—but, according to my understanding of the model, this should just be equal to x, as being solitary is independent from being fast. The authors could also consider plotting the number of solitary slow cells, solitary fast cells, grouped slow cells, and grouped fast cells over time in a single stacked area plot to make the population dynamics for insightful for the reader.

Reply: We suppose that the reviewer referred to Fig. 1. We have changed the axis as they suggested. We have also substituted Supp. Fig. 1 with the variables (numbers of cells belonging to different classes) suggested by the reviewer, that are indeed more useful for visualizing the dynamics of the population. We added as a separate supplementary figure the payoffs of fast and slow cells, and juxtaposed the trajectory of the population size as a reference for the cycle phase.

5. In Figure 3, should the y-axis for 3B say "λ_F" instead of "F"? In the inset in 3A, please indicate the direction (clockwise or counterclockwise).

Reply: We thank the reviewer for pointing out this error. The y-axis now says λ_F, and the direction of the cycle has been added.

6. Figure 4: should it be λ_S^* on the x axis? Otherwise, I don't understand how Fig. 4 relates to equation (3), which shows that S (not S^{-1}) is a linear function of λ_S. In Figure 4, what is the assumption on the mutant growth rate? S depends on both λ_S and λ_S^*, but it is plotted as a function of only one.

Reply: Thank you for this remark! You are right: the proper label should be λ_S^* indeed. In order to plot Figure 4, we consider a small fixed difference ($\lambda_S - \lambda_S^* = 10^{-1}$) and vary λ_S^*. We have added a sentence in the main text explaining this detail (lines 248-250).

7. How sensitive are the results to the assumption that the payoff of slow cells scales linearly with the fraction of fast cells?

Reply: We expect that a nonlinear monotonic payoff would change the shape of the (generic) bifurcation diagram, but not affect the qualitative results. A non-exhaustive exploration of a few nonlinear (saturating and quadratic) payoff functions did not evidence qualitative differences, nor more complex oscillations, but we cannot exclude they may take place for other functional forms or regions of the parameter space we have not sampled.
Even though it would be definitely interesting to explore different scenarios, in this work there was no biological reason to introduce nonlinearities. In order to keep the message as simple as possible (which turned out to be already a challenge), we decided to stick to the simplest version of the model.

8. I was initially confused by the model setup (1), in which the replicator equation is used to write an equation for dx/dt. It was not immediately intuitive to me how the equations for dN/dt and dx/dt would interact. More intuitive to me would be to, instead, write equations for the fast cells F and the slow cells S. These equations are simply $dF/dt = RFp_F - dF$ and $dS/dt = RSp_S - dS$. I think it would be valuable to mention this equivalent setup as well, as it allows the reader to understand the dynamics of a single cell type from a single equation (rather than having to combine the equations of dN/dt and dx/dt).

Reply: We have chosen to use the total population size and fraction of fast cells as state variables because the former directly connects to the ecological dynamics of the population, and the latter to the evolutionary dynamics, typically described by a replicator equation. We added in the Supplementary Information, Appendix A the equivalent formulation in terms of the number of fast and slow cells, and the derivation of eq. (1). This alternative formulation is now referred to in lines 96-97. The variations in the number of cells in each of the four states (fast, solitary; fast, grouped; slow, solitary; slow, grouped) are now illustrated in Supp. Fig. 1.

9. In the Introduction, the grouping mechanism (lines 76-80) should be explained better. In particular, the sentence "We model this by assuming that the probability to join a group is inversely proportional to resource density" is confusing. Perhaps better is something along the lines of "We assume that, at any point in time, the fraction of cells that is in a group (whether fast or slow) is equal to $1 - R/K$. This means that all cells are solitary when the resource is at carrying capacity, and that all cells are in groups when the resource is completely depleted."

Reply: Many thanks for the suggestion. We have now replaced the confusing sentence with a slightly modified version of the one you have suggested. See line 108-112.

10. In line 101, should "survive" be replaced by "reproduce"?

Reply: You are right. We introduced this change (line 142).

11. Lines 167-168: "Numerical simulations indicate ... it does not diverge". While numerical

simulations can be suggestive (although I am not so confident they are, in this case), they are not by themselves sufficient to claim a function diverges. This claim should be removed. I do think the authors' comments on finite populations (170—173) are intuitive and helpful; these considerations also do not depend on the exact asymptotic behavior of the period of the oscillations.

Reply: Our intention was to point to the fact that one might expect a homoclinic bifurcation, where the limit cycle would suddenly 'break' and oscillations would be lost discontinuously, to occur at high exploitation levels. Such a global bifurcation is associated to the period of oscillations having a vertical asymptote. The scaling of the period indicated that this does not seem to be the case, so that oscillations would not be lost in the deterministic system. We however decided not to go into the details of this argument and changed the sentence (lines 203-205), so that it is focused on the amplitude of the oscillations, that is indeed the determinant element of fragility with respect to stochastic fluctuations.

In order to avoid repetitions (point 20) we moved the discussion on the effect of finite-size fluctuations entirely in the discussion (lines 345-347).

12. Consider rewording lines 138-140. Saying that slow cells spread within aggregates can be misleading, because the fraction of slow cells that belong to aggregates is constant.

Reply: We have reworded the sentence relative to the spreading of cheating (line 181).

13. In line 180, "the time-scale separation is highest close to the onset of the oscillations." I don't understand how the authors get this from Figure 3. For example, if I take $\lambda_F = 10$ and increase λ_S, the period of oscillations only increases.

Reply: The timescale separation between the life-like cycle and the cell duplication rate (maximal or averaged over a cycle) is illustrated in (current) Supp Fig 5 and Supp Fig 6, and not in (current) Supp Fig 4. To avoid confusion, we have repeated the reference to those figures at the end of the sentence.

14. Line 182: remain -> remains.

Reply: Changed.

15. Line 210, what is meant by "the dominant Lyapunov exponent 'transverse' to the resident limit cycle"?

Reply: The Lyapunov exponent transverse to the limit cycle measures the stability of the limit cycle in a direction orthogonal to the limit cycle itself, like that associated to a newly introduced mutant. Since in our context the importance of this notion lies in the fact that a perturbation grows - or not - we have removed this precision. We now just explain it (lines 253-254) in terms

of the average growth rate of a mutant (one has to take a time average because, being on a limit cycle, the trajectory is oscillating around an exponentially varying trend).

16. In line 216, "the emergence of a new collective time scale constrains its effects to a single phase of the life cycle". Is this really true? Cheating is always constricted to the group phase of the life cycle by the way the model is constructed.

Reply: Yes, but its effects are not felt when cells are not aggregated. It is the presence of a social, aggregated phase that makes cheating important. As long as cells remain mostly solitary, social conflicts are there, but their effects are small.
We have rephrased the sentence so as to make this more clear (lines 261-263).

17. The suggestion in lines 219-229, that slow-down of evolutionary dynamics at high levels of cheating may be a mechanism for coexistence, I found quite enticing.

Reply: Many thanks for the comment.

18. Mutations are required to transition between fast and slow cells. There may be interesting parallels with Paul Rainey's work on the emergence of multicellular life cycles, because the life cycles in his work also initially depend on mutations (e.g. Hammerschmidt et al. 2014). The discussion in lines 280-282 could be expanded if the authors think these parallels make sense.

Reply: The work of Paul Rainey is indeed relevant to the discussion of how alternating selective pressures can lead to the evolution of phenotypic multistability. We have added this citation as an example of how the existence of a collective timescale can select for what most people would be comfortable to call a life cycle (lines 27-30 and 302-308).
However, the mutation from fast to slow cells is not (yet) included in our model, as was pointed out by reviewer 3. The mutations that we consider in the adaptive dynamics model are such that slow cells change their exploitation level, not their velocity. It is however certainly interesting to conceive the possibility that cells switch from fast to slow and vice-versa. As long as the transition rate is small, the additional term that would appear in the frequency equation (a constant additive term) should not alter qualitatively the results. What would be more interesting, is to let such rate depend on the context, but that would complexify the model further.

19. The discussion of parallels with D. discoideum (paragraph starting at line 284) belongs in the Introduction, because these observations serve as motivation for the model.

Reply: We moved that information to the introduction, where we motivate the relevance of considering slow and fast cell types (lines 37-43). We left nonetheless in the discussion the observation that our model naturally provides a temporal (or developmental) dimension also to the inter-group social dynamics between collective generations, that is missing in many game-theoretical models (lines 328-333).

20. Line 300-303 add little beyond what is said in the Results section, and can be removed.

Reply: We deemed useful to discuss stochastic fluctuations together with other ways of making the model more realistic. We have therefore removed the comment on the effect on high-amplitude oscillations from the results section, in order to avoid repetitions (see point 11).

Reviewer 3

The authors provide an explicit scheme in which a spontaneous transition occurs from a unicellular to a multicellular stage and back. The paper deals with the maintenance of a uni+multi-cellular life cycle, not to its emergence, and some details are omitted or obscure. Those negative comments notwithstanding, the paper marks a significant advance in the modeling of life cycles. For that reason, it deserves to be published, but after revision.

Reply: We thank the reviewer for their overall positive assessment. We realized that a number of modelling choices and interpretations were not made sufficiently clear in the manuscript, and we explicited our reasoning, as detailed below and in the answers to Referee 2.

General remarks

In essence, the model postulates cellular heterogeneity, two life cycle stages in which one cell type gains a fitness advantage over the second and vice-versa, and a coupling to nutrients that are renewed substantially during one of the two stages (during which feeding is diminished). No aspect of the model deals with motility differences per se, or, for that matter, with movement: the life cycles are purely temporal and play out in a spatially well-mixed system. The terms "fast" and "slow" have been grafted on to the model to lend it biological plausibility. One can think of traits other than speed of movement that could have served the same purpose.

Reply: It is true that we expect oscillations of a similar kind to be possibly generated when trade-offs between the solitary and aggregated phases of the cycle stem from mechanisms other than motility. As we argue in the introduction, however, we think that motility is particularly important because: 1) it naturally provides both a mechanism for aggregation and it underpins collective motility, that is the public good and a distinctive feature of aggregative life cycles in Dictyostelids and Myxobacteria; 2) motility differences are naturally related to cell sorting, so that they produce 'for free' the geometric organization of Dictyostelium slugs (as proposed by Bonner 1957); 3) motility is usually considered as a hindrance to the evolution of cooperation, because it enhances mixing and reduces relatedness (as argued also in Matapurkar and Watve 1997).
We have modified the introduction in order to make these points more clear (lines 36-62).

We would also like to stress, in relation to a later point, that there are no pre-existing 'phases' of the life cycle when nutrients are repleted or dispersal imposed (as in previous models). Such phases can be recognized once the oscillations get established. In the equilibrium solution, there is none, which proves that they are not a trivial consequence of the assumptions of the model.

Importantly, the differences between the two types is heritable, meaning that the differences involve two genotypes. This point should be clarified. In the absence of any reference to genetic heterogeneity in multicellular groups, and given that metazoans are believed to be clonal, it lessens the biological relevance of the model. It would have been more interesting to deal with clonal cells and alternative phenotypes, and the authors may have an extension in mind.

Reply: This point rejoins the criticism on the 'life cycle' interpretation. We agree that this difference with respect to life cycles – that cells undergo a population dynamics instead of changing their phenotype – is important. We had decided to stress the analogies with aggregative life cycles: the existence of a cycle, the ordering of the phases, and the conflicts experienced within heterogenous aggregates, but we have now reformulated the title and the phrasing in the results, stressing that the heterogeneity of the aggregates is a key point.
We still maintain that the temporal structure that characterizes these eco-evolutionary cycles is crucial to understand how a cycle that comprises starvation-induced aggregation and disaggregation gets established in the first place. These cycles are fundamentally different from paradigmatic life cycles with clonal growth, exactly because aggregation entails mixing of genetically different cell types. We therefore refer to the cycle as a 'life-like (aggregative) cycle', and stress that it provides a timescale for the aggregation dynamics, which is exactly what previous models presupposed.
In the discussion (lines 298-302), we have expanded on the possibility that the life-like cycle may act as a scaffold to the evolution of regulated phenotypic switches, and refer to previous theoretical and experimental results obtained by imposing extrinsic periodic variations in selective pressure.

As mentioned, the model does not deal with emergence at all. I suggest changing the title to something that correctly reflects the content of the paper, e.g., "The evolutionary stability of life cycles with heterogeneous cell types". Differential motility is certainly an interesting idea, and the Discussion can touch on it, and other candidate traits (e.g., adhesion), as ways to instantiate heterogeneity.

Reply: We contend that the term emergence is well suited for the phenomenon we study. Indeed, oscillations and 'cycle phases' only occur in certain circumstances. Interestingly, such circumstances are achieved when the social conflicts worsen and the collective function gets increasingly exploited. The evolutionary dynamics drives the system through a bifurcation, so that the cycle literally only exists after the transition occurred.
We nonetheless changed the title in order not to refer to life cycles.
We have expanded the introduction so as to explain why we think that motility is an interest-

ing factor to take into account, but now the discussion also mentions the possible role of other sources of phenotypic heterogeneity (lines 334-340).

Model

(I have not checked all the mathematical results and have taken many as valid because the results look plausible.) One feature of the model is puzzling. I can understand that the probability of being solitary should be positively correlated with R, the amount of available food resource. But why is it negatively correlated with K, which is the carrying capacity of the environment? What makes a cell sensitive to how much of the resource can be sustained by the environment?

Reply: This choice just reflects the fact that it seemed easier to express the payoff in terms of probability of being aggregated. As a probability cannot be larger than 1, we normalized by K. A different choice, where we would consider the rate instead of the probability, would only differ in an arbitrary factor in the parameter λ_F. As long as K is kept constant, as we do, there is no difference in the two formulations. We decided to keep our first formulation, but added a comment on the generality of the choice (lines 127-128).

Minor points

Justify the analogy to public goods games - what is the public good here?

Reply: The public good is the advantages conferred to the aggregate by collective motion, thanks to the presence of fasts cells. The parallel between fast motion and cooperative behaviour is based on the observation that in Dictyostelium fast cells are mostly found on the pre-stalk region of the slug. More generally, if collective displacement has – as often assumed – a function, than fast cells can be expected to carry most of its burden, and slow cells to exploit it.
We have modified the introduction (lines 40-43) and the results (lines 133-134) so as to make this point more clear.

In Appendix A, just before eq.(4), it says "Eqs. 10- 12 has one fixed point", presumably meaning eqs. 1-3.

Reply: Well spotted, the reference has been fixed. Many thanks for pointing this out.

The statement "Motility alone is sufficient to give rise to aggregates that can be long-lived [15, 16]" is misleading. Crucially, in addition to motility, those works assume that a cell adopts the average orientation of its neighbors.

Reply: We have rephrased the sentence and provided a reference to a work where no alignment (nor adhesion) is assumed (lines 44-46). The fact that moving objects form clusters when their

density is sufficiently high is however a phenomenon known for many self-propelled particles models and a variety of interaction types (chiefly adhesion and/or alignment). We did not wish to enter in these distinctions here, also because differences between models with different assumptions are not fully worked out, but rather to point out that aggregation – though not aggregation cycles – occur generically in motile cells.

With regard to the comparison with Dictyostelium, there are two papers that overlap with the present one. In "A Theory of the Control of Differentiation in the Cellular Slime Molds" (Quart Rev Biol 32(3): 232-246, 1957), J. T. Bonner proposes a model for differential motility leading to multicellular organization in Dictyostelium. Also in the context of Dictyostelium, Matapurkar and Watve show in a computational model that it is possible to get stable oscillatory coexistence of two behavioral cell types that originate de novo in each life cycle (Altruist cheater dynamics in Dictyostelium: aggregated distribution gives stable oscillations, Am Nat 150(6):790-797, 1997). The authors should mention both and point out in what respects their model is different.

Reply: We thank very much the reviewer for pointing out these very relevant papers. We have included the first to support our interpretation of fast cells as 'cooperators' (lines 307-308). We comment the second in the discussion on lines 335-340.
