# Supplementary material for: Aggregative cycles evolve as a solution to conflicts in social investment
Source: PLoS Comput Biol. 2021 Jan 20;17(1):e1008617. doi: 10.1371/journal.pcbi.1008617 (PMC7850506; doi:10.1371/journal.pcbi.1008617)
Supplement: Attachment — Submitted filename: Response to reviewers.pdf [file pcbi.1008617.s012.pdf]

Dear Dr. Tarnita,

we thank you and the two reviewers very much for the insights you brought, that helped us a lot in understanding what was missing in our work.
We have made additional changes to the manuscript (marked in the resubmitted manuscript) so as to respond to the referees comments.
Please find below our detailed answers to the additional points raised by the referees.

With best regards,

Leonardo Miele and Silvia De Monte

Reviewer 2

I reread the revised version with interest and I believe the manuscript has substantially improved. I found that the presentation and the more conservative interpretation of the emergent cycles as "life-like cycles" rather than "life cycles" do more justice to the interesting results in this paper, which I (even more so when reading this paper again in its revised form) found fascinating and thought-provoking. In sum, the revised version was, at least for me, much more effective in driving home what makes the results of this paper novel and interesting. I'm also happy with the authors' detailed and thoughtful responses to my comments, including those that challenged the authors' interpretation and those that arose from a misunderstanding on my end. I only have a small number of remaining comments and suggestions.

Reply: We thank the reviewer very much for their appreciation. We also feel the manuscript has improved thanks to the feedback received. We have made additional changes in order to answer remaining questions.

1. In the abstract, and the discussion, the author write the emergent oscillations are an "adaptive response to [the escalation of conflicts]". I'm not sure what to make of this claim. Adaptive to whom?

Reply: We changed the sentence so that it is clear that the onset of oscillations is the consequence of selection for cell traits.

2. Line 5: transition -> transitions.

Reply: Changed, many thanks for that.

3. Line 30 could perhaps be more compelling if this paragraph on previous work ends by explicitly stating what is missing from previous work

Reply: We have rephrased that sentence and slighly modified the following one in order for the transition to be more explicit.

4. *Over all I found the explanation in the introduction very clear.*

5. *Line 101: context -> social context.*

Reply: Changed.

6. *Line 124-125: I was thrown off by the added statement in the parentheses. I noted that this was in response to another reviewer's comments, but it's not clear what the "without loss of generality" statement applies to.*

Reply: Indeed, we realized that what we thought was a simple parameter rescaling is actually more complicated. Thank you for drawing our atention to this point. See response to referee 3 for details.

7. *In the caption of Figure 2, mention what the dashed line is.*

Reply: We have added the following sentence to Fig. 2 caption "Dashed lines indicate the position of the unstable co-existence equilibrium (analytically derived in the SI)."

8. *Line 196: is -> are.*

Reply: Changed.

9. *Line 197: Here a reference to "life cycle" remains.*

Reply: Changed.

10. *Line 216: Similarly, I think here the authors want to say "life-like cycle".*

Reply: Changed.

11. *Line 330: cell -> cells.*

Reply: Changed.

Reviewer 3

Most of my comments have been addressed satisfactorily. Now I understand what the authors mean by "emergence"; it is the emergence of a life cycle, not emergence of multicellularity, which was my impression at first. Incidentally, unlike the other reviewer, I see no objection to using "life cycle". Indeed, "life-like cycle" sounds rather awkward. There are three points which I would like the authors to consider once again, but do not insist that they accept my advice.

Reply: We thank the reviewer for their insightful comments. We have taken them into account in preparing the final version of the manuscript. However, we decided to stick to the notation life-like cycle in order not to raise misunderstandings as to the nature of the changes in the population's phenotypic composition.

(a) I agree that motility is an excellent candidate for the basis of what makes the two cell types different. Also, as the authors rightly say, it strengthens the comparison with the documented Dictyostelium example. But my point was that motility plays no role in the model. Ipso facto, motility differences play no role either. The model demonstrates how temporal oscillations in a well-mixed heterogeneous system, along with two time scales, can emerge as an automatic consequence of three factors: the dynamics of growth, resource level-dependent aggregative behaviour, and frequency-dependent fitness. That being so, the use of "motility" in the title (now amended to "Aggregative cycles evolve as a solution to conflicts in motility investment") is not correct. A more appropriate title should be chosen. The text can remain unchanged, except that a sentence can be added stating that motility is a plausible candidate for the differential phenotypic trait they are considering.

Reply: We changed the title in order to stress that the important ingredient is that evolving traits are linked to social conflicts. We also added a sentence in the introduction stressing that our focus on motility stems from the need of making realistic hypotheses on the rates at which individuals join groups and contribute to group function.

(b) The factor R/K appears naturally in the equation $dR/dt = R[r(1-R/K) -N]$, because it shows that the intrinsic rate of growth of the resource is zero when the carrying capacity of the environment has been reached. However, it is a different matter to say that the same R/K is also the probability that a fast cell remains unaggregated, because that gives R/K an additional role in the model. It affects the payoffs, and therefore the dynamical consequences of equations (1). The authors say that the 1/K factor is used simply to make R dimensionless, and any other factor with the same dimension as 1/K would do. They may be right. But I am unable to see it immediately. Readers will benefit if the authors provide an explicit demonstration in the supplementary file. The text can just make a mention of it.

Reply: We realized that indeed, as the reviewer intuited and contrary to what we thought, when the maximal probability of aggregation (attained when the resource reaches its carrying capacity) is not 1, a further independent parameter is introduced in the equations. We have studied the

bifurcation diagram in this case and found that the results are not qualitatively affected, though the bifurcation curve displaces towards larger values of both λs when the aggregation probability decreases. We have added a section in the SI discussing this point, and rewritten the main text so as to indicate this additional information. Thank you for pointing this out repeatedly, and sorry for having overlooked your previous comment.

(c) The description of cell speed as a public good continues to bother me. The authors explain it as the benefit conferred by fast cells to slow cells that are in the same collective. It seems to me that in order to qualify as a public good, the same benefit must be available to other fast cells in the group too. It would amount to saying that a collective made up only of fast cells will move faster than any fast cell could by itself. That is plausible as a correlate of the size-dependence of speed, as found in the case of Dictyostelium slugs (Inouye and Takeuchi I 1979 Analytical studies on migrating, movement of the pseudoplasmodium of Dictyostelium discoideum; Protoplasma 99: 289-304). The authors could make use of this finding to justify their assumption. But it would still not qualify for the designation of public good.

Reply: It is not cell speed that is the public good, but the collective displacement that is possible thanks to individual cells aligning their motion. Collective displacement is commonly assumed to be a function, achieved through aggregation, of the multicellular collective, e.g. Dictyostelium slugs. The fact that faster collective motion, enabled by a higher fraction of fast cells, provides increased benefits is also commonly hypothesized in models for the evolution of cooperation in multicellular aggreagates, e.g. in the paper of Matapurkar et al. or of Guttal Couzin. We therefore do not think that this modelling assumption requires additional discussion.
