# Supplementary material for: Aggregative cycles evolve as a solution to conflicts in social investment
Source: PLoS Comput Biol. 2021 Jan 20;17(1):e1008617. doi: 10.1371/journal.pcbi.1008617 (PMC7850506; doi:10.1371/journal.pcbi.1008617)
Supplement: Attachment — Submitted filename: REPORT - Miele - De Monte - Evolution of aggregative life cycles.doc [file pcbi.1008617.s010.doc]

Comments on “The evolutionary emergence of aggregative multicellular life cycles” (Miele and De Monte, PCOMPBIOL-D-20-01189)

The authors provide an explicit scheme in which a spontaneous transition occurs from a unicellular to a multicellular stage and back. The paper deals with the maintenance of a uni+multi-cellular life cycle, not to its emergence, and some details are omitted or obscure. Those negative comments notwithstanding, the paper marks a significant advance in the modeling of life cycles. For that reason, it deserves to be published, but after revision.

*General remarks.* In essence, the model postulates cellular heterogeneity, two life cycle stages in which one cell type gains a fitness advantage over the second and vice-versa, and a coupling to nutrients that are renewed substantially during one of the two stages (during which feeding is diminished). No aspect of the model deals with motility differences per se, or, for that matter, with movement: the life cycles are purely temporal and play out in a spatially well-mixed system. The terms “fast” and “slow” have been grafted on to the model to lend it biological plausibility. One can think of traits other than speed of movement that could have served the same purpose.

Importantly, the differences between the two types is heritable, meaning that the differences involve two genotypes. This point should be clarified. In the absence of any reference to genetic heterogeneity in multicellular groups, and given that metazoans are believed to be clonal, it lessens the biological relevance of the model. It would have been more interesting to deal with clonal cells and alternative phenotypes, and the authors may have an extension in mind.

As mentioned, the model does not deal with emergence at all. I suggest changing the title to something that correctly reflects the content of the paper, e.g., “The evolutionary stability of life cycles with heterogeneous cell types”. Differential motility is certainly an interesting idea, and the Discussion can touch on it, and other candidate traits (e.g., adhesion), as ways to instantiate heterogeneity.

*Model.* (I have not checked all the mathematical results and have taken many as valid because the results look plausible.) One feature of the model is puzzling. I can understand that the probability of being solitary should be positively correlated with R, the amount of available food resource. But why is it negatively correlated with K, which is the carrying capacity of the environment? What makes a cell sensitive to how much of the *resource* can be sustained by the environment?

*Minor points.*

Justify the analogy to public goods games - what is the public good here?

In Appendix A, just before eq.(4), it says “Eqs. 10- 12 has one fixed point”, presumably meaning eqs. 1-3.

The statement “Motility alone is sufficient to give rise to aggregates that can be long-lived [15, 16]” is misleading. Crucially, in addition to motility, those works assume that a cell adopts the average orientation of its neighbors. With regard to the comparison with Dictyostelium, there are two papers that overlap with the present one. In “A Theory of the Control of Differentiation in the Cellular Slime Molds” (Quart Rev Biol 32(3): 232-246, 1957), J. T. Bonner proposes a model for differential motility leading to multicellular organization in Dictyostelium. Also in the context of Dictyostelium, Matapurkar and Watve show in a computational model that it is possible to get stable oscillatory coexistence of two behavioral cell types that originate de novo in each life cycle (Altruist cheater dynamics in Dictyostelium: aggregated distribution gives stable oscillations, Am Nat 150(6):790-797, 1997). The authors should mention both and point out in what respects their model is different.
